# American Dental Convention

**Published:** 1864-10

**Authors:** G. W. Ellis


### AMERICAN DENTAL CONVENTION.

BY G. W. ELLIS, M. D.

The Convention assembled on Tuesday, August 2, 1864, in the Supreme Court Room, Detroit, Michigan, and was organized at nine A. M., with the President, Dr. Taft, in the Chair.

Drs. Whitney and Benedict were appointed to assist Dr.

Watling in discharging the duties of the Executive Committee, and after consultation, one dollar was announced as the individual assessment, when the following gentlemen came forward, paid their contributions, and signed the Constitution:

Illinois.—W. H. Chaffee, Alton; J. Ward Ellis, T. P. Abell, W. W. Allport, S. B. Noble, L. P. Haskell, Chicago.

Indiana.—W. F. Morrill, New Albany.

Iowa.—W. O. Kulp, Muscatine.

Kentucky.—H. McCollum, Augusta.

Massachusetts.—S. G. Henry, Westboro'; H. F. Bishop, Worcester.

Michigan.—G. B. Cady, M. H. Knapp, Adrian; G. W. Stone, Albion; C. B. Porter, Ann Arbor; J. A. Robinson, G. H. Mosher, Jackson; Isaac Douglass R. S. Bancroft, Romeo; J. A. Watling, S. A. Gerry, A. F. Barr, Ypsilanti; L. A. Rogers, J. C. Parker, Grand Rapids; J. A. Harris, Isaac Voorhies, Pontiac; John H. Warner, St. Clair; S. H. Burgess, Owasso; Jared Kibber, Port Huron; Geo. H. Cooper, Thos. A. White, J. H. Farmer, Henry Cowle, Geo. L. Field, H. Benedict, Detroit; C. E. Bartlett, Battle Creek; H. Cole, Grand Ledge; E. Hause, Tecumseh; H. H. Jackson, Northville; Geo. E. Corbin, St. Johns; W. C. Britain, Birmingham; L. C. Whiting, East Saginaw.

Missouri.—C. W. Spalding, Isaiah Forbes, W. N. Morrison, St. Louis.

New York.—W. H. Atkinson, W. H. Allen, New York City; B. Wood, J. A. Perkins, Albany; B. T. Whitney, Geo. E. Hayes, Buffalo; P. Harris, Skaneateles; D. L. Overholser, Lockport; F. M. Briggs, Stockton; H. Jameson, Jr., Lyons; J. Naramore, Rochester; S. W. Robinson, Watertown.

Ohio.—J. Taft, P. Knolton, Cincinnati; E. E. Rogers, Hudson; W. King, Berg Hill; G. W. Nelson, Ashtabula; W. E. Dunn, Delaware.

Pennsylvania.—Geo. W. Ellis, T. L. Buckingham, C. N Peirce, J. R. McCurdy, Thos. H. Stockton, Jr., S. S. White.

Philadelphia; B. M. Gildea, Harrisburg; J. B. Williams, Monongahela City; W. E. Magill, Erie.

TENNESSEE.—S. J. Cobb, Nashville.

CANADA.—Geo. Shattuck, Tillsonburg; L. Clements, Kingston; J. Bowers, Ingersoll; F. G. Callender, Coburg; W. W. White, Chatham.

The minutes of the preceeding session were read and adopted.

The Executive Committee selected and submitted the following subjects for discussion, their report being adopted:—

ORDER OF DISCUSSION.

1. The best means of improving the practice and elevating the profession of dentistry.
2. Anæsthetics—their proper use and relative value.
3. Extracting Teeth—when it should be done and when not; the best instruments for the purpose, and the subsequent treatment, when any is required.
4. Absorption of Alveolar Process—Causes and treatment.
5. Filling Teeth—the relative value of different materials, and the mode of operating in difficult cases.
6. The best method of obtaining accurate impressions and models of the mouth.
7. The relative value of different materials as a base for artificial teeth.
8. Miscellaneous.

L. W. ROGERS, Utica, N. Y.
A. W. KINGSLEY, Elizabeth, N. J.
J. A. WATLING, Ypsilanti, Mich.
A. HILL, Norwalk, Conn.,
H. A. SMITH, Cincinnati, Ohio,
} *Executive Committee.*

The Committee on the preparation of the Kingsley Medal asked further time, which was granted.

The Treasurer presented the following statement:—

| | |
|---|---|
| Received from former Treasurer | $62 69 |
| From annual assessments | 103 00 |
| Total | $165 69 |
| Expended | 151 75 |
| Balance | $13 94 |

DRS. W. H. ALLEN, MCCOLLUM, and GILDEA were appointed an auditing committee. Upon examination, the report was found correct, accepted, and the committee discharged.

The committee on the admission of dentists into the army was reported by Dr. S. S. White, who repeated the statement made before the Association at Niagara;

The report was accepted and the committee continued.

DR. WHITNEY offered the following:—

*Resolved*, That this Convention extend an invitation to the medical profession, members of the press, and all others interested, to attend its sittings. Carried.

DR. WHITNEY moved that the Convention hold two sessions, from nine A. M. to half-past twelve P. M., and from half-past two to six P. M.

DR. PERKINS amended so that the morning session commenced at eight A. M.

DR. ELLIS thought that if the time for final adjournment be fixed, much more would be accomplished in the same period than if it were left undetermined. He therefore offered the following amendment: "and that the final adjournment take place at 5 P. M. on Thursday."

The resolution, thus amended, was carried.

The election of officers being in order, Drs. Kulp and Bishop were appointed tellers.

The ballotting resulted in the unanimous choice of the following officers:—

*President.*—DR. W. W. ALLPORT, Chicago, Ill.

*Vice-President.*—DR. H. F. BISHOP, Worcester, Mass.

*Recording Secretary.*—DR. G. W. ELLIS, Philadelphia, Pa.

*Corresponding Secretary.*—DR. W. H. ALLEN, New York, City.

*Treasurer.*—DR. H. BENEDICT, Detroit, Mich.

DR. BUCKINGHAM moved that the choice of the next place of meeting be made the special order of business for three P. M. the following day.

This was amended to twelve M., and so adopted.

Upon motion, the consideration of subjects five and six upon the order of discussion were reversed.

The induction of officers being in order, Drs. Spalding and Forbes were appointed a committee to conduct the President elect to the chair, who, upon assuming the position, returned thanks for the honor conferred, and remarked that it was not his intention to make a speech. He deemed it the duty of the presiding officer to facilitate business and set a good example. How far he should succeed he could not say, but would endeavor to merit approbation.

There being no miscellaneous business, adjourned to half-past two P. M.

FIRST DAY.—*Afternoon Session.*

Called to order at half-past two P. M. The minutes of the previous session were read, and, after some minor modifications, adopted.

The retiring President, DR. TAFT, said that he was desirous of making but a few remarks. He spoke of dentistry as a modern institution, partaking of the characteristics of the age, and combining, in an admirable degree, utility, growth, vigor, energy, and a capability of accommodating itself to circumstances. He said that a retrospect, carrying us back to hold converse with the pioneers and founders of that profession which we view with so much pride, would prove eminently pleasing and instructive, and admiringly referred to the efforts of a Harris, Kœcker, Greenwood, etc. He spoke of the great progress of the present, and instanced the perfection of artificial work, in beauty, serviceability, power of restoring contour, and improving speech accomplished by an Allen, Kingsley, and Austin; the advanced standard of pathology borne by an Atkinson, Spalding, etc., and the host of able and earnest workers in the operative department. He said that everything which stands the test and proves truly valuable comes only as the result of labor; he would therefore urge all to renewed effort, deeming it the province and duty of the most advanced to go still further, while assisting those below them up to a more elevated position. He regarded dentistry as a humane profession, and thought that at the present day the public were privileged to *demand* relief from suffering and disease; and any man who does not love his specialty, or follows it from sordid motives alone, has mistaken his calling, and should correct his error. He had heard the dignity of the profession frequently spoken of and its injury prophesied and apprehended; yet he thought it useless to dwell upon such a subject, when, by the performance of individual duty, its establishment and maintenance were certain. He regarded associated effort as a powerful agency for advancement, and viewed with pleasure the promising future.

The first subject, "The best means of improving the practice and elevating the profession of dentistry," was declared in order.

Dr. Taft spoke of the mean character of that competition which reduces the standard of work, both in price and quality, and regarded it the duty of every practitioner to execute the best operations, employ the best instruments, material, etc., and charge accordingly, believing it an impossibility to elevate the profession without first improving the practice. He referred to the necessity of study and investigation to insure advancement, and believed that the continuation of such progress as we now witness would in a few generations do away with the necessity for artificial dentures.

Dr. Atkinson said the question was one which had long been of interest to him. The reason why a profession commands more than any mere handicraft is, because of the time and labor spent in the acquisition of the knowledge necessary for its execution; and in consideration of this fact, he regarded it a shame that the colleges were so poorly supported, and thought there should be encouragement enough offered to lead to the establishment of a school in every State, instead of the meager patronage accorded to those already in existence. He considered that dentists should be anatomists, chemists, metallurgists, and the embodiment of a thorough knowledge of all the physical sciences; and in view of such a vast field, would oppose any admission into the ranks of our profession without the evidence of an initiatory pupilage, of at least three years. He thought every one should recognize his own and others' powers and shortcomings, and evince a willingness to occupy his own sphere, and accord to each the position which his qualifications merit. He strongly favored association, and believed that exclusiveness would lead to retrogression.

Dr. Spalding thought that an effort for the elevation of the profession should be preceded by an investigation of the depressing influences. He thought it a prime defect to permit the student to enter upon the study of dentistry, without first impressing the absolute necessity of a good education, the magnitude of the task he proposes to assume, and the sacrifices required in the execution of its demands. Another improvement, capable of elevating the status of the profession, is the education of the people, a measure he would advance by any legitimate means.

Dr. Perkins thought the remuneration a very important matter, not to be ignored, since each man is supposed to have chosen his occupation as the means of procuring himself a livelihood. He advocated earnestness in the investigation of any subject, and while he was capable of enjoying a good joke, would at such times discountenance all frivolity.

Dr. Magill advocated the education of the people through the agency of the press, and considered the performance of good work almost as available for imparting a knowledge of the importance and value of dentistry. He hoped by such means to realize the proud position to which we so earnestly aspire. He believed that circumstances would vary charges, and thought that in some cases the remuneration offered would not command the highest class of work.

Dr. Peirce believed with Dr. Taft, that we should not endeavor to make work meet a pre-established fee, but should charge in accordance with our estimate of its value when completed. He condemned discrimination between rich and poor, and would do his best for the servant as well as for the master.

Dr. Bishop always prepares the mind of the patient during the operation, by conversation, from which by inference the cost of the work can be approximately anticipated. He thought that much valuable information could be imparted by conversation at the chair, and regarded this as one of the easiest and most available means offered for the education of the public.

Dr. Robinson remarked that in saying it is the *quality* of the work which makes the man, he but reflected the minds of all present. He had removed to Michigan some years since, and although at first opposed in his prices, he soon surmounted the difficulty; as any one may do who is willing to be estimated by his works.

Dr. Spalding can not fix the price of an operation before its performance. He would, however, give an approximation of its value, believing that much due the patient.

Dr. Perkins advocated giving the patient an approximate of the cost of operation, in order to avoid any dissatisfaction which might arise from an unexpected heavy charge. Thought that such a course would avoid misunderstandings, and establish feelings of friendship and appreciation.

Dr. Atkinson thought that the propriety of such a course depended upon circumstances, and advocated every practitioner taking a high moral position, and thus teaching others to rely upon his honor.

DR. MAGILL, speaking in relation to high prices, advocated in dentistry, as in any other occupation, the greatest good to the greatest number; and believed that any philanthropic and honorable practitioner would serve the poorer class, rather than force them into the hands of charlatans.

DR. PERKINS does not advocate exorbitant charges, but fair remunerative prices.

DR. McCOLLUM said that every man should charge in accordance with the estimate he places upon his own ability, and the public will be found to place a like estimate upon his acquirements.

DR. BUCKINGHAM moved going on to the next subject, "Anæsthetics—their proper use and relative value."

A paper from Dr. G. T. Barker, upon Anæsthesia, was read by the Secretary. Dr. Atkinson also read a paper upon the same subject.

DR. BUCKINGHAM objected to the synonymous use of words and expressions, which convey to the mind different impressions, and considered it a mistake to confound, as had been done, the galvanic and magnetic forces with life force. He described the difference between the animal and vegetable cell, and did not think that either were oxidized or deoxidized, or contained perceptibly more oxygen one time than another; but if all action is arrested, anæsthesia will be induced.

DR. GERRY could not understand the theory of polarization but had experimented with chloroform to a great extent, and found that its first effect was to lull the nerves of sensation, and afterward those of motion. He never saw a case of death resulting from its administration, except among the inferior animals, of which he had killed a host, having exhibited it to over four hundred; a plan which he advocates as invaluable for the acquirement of information. He would not decide in regard to the relative safety of different agents; but himself accorded the preference to chloroform. He believed the medulla almost the first point touched, as evidenced by the disturbed respiration. In its administration, he prefers the recumbent posture, as anæsthesia is more readily induced. When it is hastily given, death is produced as it were by suffocation; but when more slowly exhibited, the effects are manifested more slowly.

Dr. Buckingham said that the effects of agents are made known only by experiment, and thought that death might be occasioned in various ways, either by over-stimulation, depression, or by direct action upon the nerves. Dr. Snow found that when the effects were very slowly induced, the animal might be revived; when, however, greater proportions were used, the heart was paralyzed, instant death resulting. He believed chloroform or ether safer than nitrous oxide gas, and urged, as objections against the latter, the difficulty of pure generation and keeping, and the method of administration.

Dr. Spalding prefers chloroform, because he knows how to use it, and advises its administration, largely diluted with atmospheric air.

Dr. Magill believed that fatal cases occurred as the result of ignorance or carelessness.

Dr. Corbin thought that none of these materials could be used with impunity, believing any agent capable of suspending sensation dangerous to a certain extent.

Dr. Spalding gives a liberal dose of some alcoholic beverage before the administration of an anæsthetic.

Adjourned.

SECOND DAY.—*Morning Session.*

Called to order at eight o'clock. Minutes read and approved.

Dr. Forbes gives chloroform to any patient, regulating the quantity according to the temperament; giving less to persons of full habit and short neck. He can not tolerate ether, and consequently never employs it.

Dr. G. W. Ellis had considerable experience with both ether and chloroform, using the former almost exclusively, but employing the latter whenever circumstances indicated. He administers it from a sponge, held in the hollow of the hands, and believed it possible, for one conversant with the anatomy and physiology of the nervous structures, to recognize each step of the operation, and trace the successive influence of the anæsthetic upon the various portions of the brain. He never employs the ether and chloroform mixed, but thinks he derives better results from their alternation in cases where it is necessary to hasten anæsthesia, or control the excessive stimulation, sometimes, though *very* seldom occasioned, by the former agent. He believed all anæsthetics dangerous in uneducated and incompetent hands, and regarded the consciousness of knowledge of the subject the true secret of success in their administration ; for one lacking faith in his own ability, can not inspire in another that confidence which is an indispensable prelude to a satisfactory result. He had no knowledge of nitrous oxide, other than that derived from reading, and the two agents mentioned proving all he desired, there had been no incentive to induce him into a practical examination of its merits. With others, he was able to say that, in his practice, extraction was now an exception, where once it approached too closely to a rule.

Upon motion, proceeded to the next subject, "Extracting Teeth: when it should be done and when not—the best instruments for the purpose, and the subsequent treatment, when any is required."

Dr. Magill, for the arrest of hæmorrhage, would first employ the simpler means, afterward resorting to a cone-shaped plug of leather. He mentioned a friend, who had met with success by the use of cowebs introduced into the socket.

Dr. Bishop wanted comparisons with regard to the general extraction of teeth, and not particularly as practiced between the ages of ten and twenty years.

Dr. Perkins mentioned a practitioner who, upon discovering decay between the bicuspids on either side, would remove one, as in attempting to save both, the two were mostly lost; this, however, was contrary to his own practice.

Dr. Robinson mentioned the case of a young man, in whom the loss of a tooth from the lower jaw, right side, and one from the upper jaw, left side, had occasioned a moving of the teeth in opposite directions, giving the jaws a twisted appearance. He asked whether, in such cases, it would be advisable to remove a tooth from the opposite side, in each maxilla, in order to prevent the deformity? He appreciated the value of the six year molars, and lamented the fact that, at ten years of age, they were mostly past redemption. He referred to a wisdom tooth which had baffled his efforts for its removal.

Dr. Bishop was in favor of putting forth every effort for the salvation of the six year molar; but regarded the bicuspids as the teeth most difficult of preservation.

Dr. Taft regretted the tendency to run into methods and rules, rather than depend upon broad principles, which alone should be discussed, and the application of which must be regulated by circumstances. He believed that teeth, which might with propriety be extracted by one operator, it would be the duty of another, more capable, to save.

Dr. J. Werd Ellis thought it important to know how to treat the teeth of children, under ten years of age, and was in favor of the preservation of those belonging to the deciduous set as long as possible. He often finds it necessary to remove sound teeth in order to afford more room in a crowded arch, or for the correction of an irregularity. When efforts for the preservation of a tooth had failed, he would advise its extraction.

Dr. Peirce thought it wrong to prematurely sacrifice either the temporary or permanent teeth, and believed that from the moment of eruption, they require the inspection of a dentist. He deemed it the duty of a practitioner to institute a close examination into the condition of the temporary teeth, and carefully fill those giving the first indication of caries. He dwelt upon the importance of preserving even the fangs of temporary teeth, in order to insure the full development of the arch; and mentioned the case of a young girl, sixteen years of age, who still retained eight deciduous teeth in the lower jaw, in addition to twelve of the permanent set.

Dr. Magill believed that the extraction of temporary teeth would sometimes injure, or even entirely destroy the permanent ones beneath, and was strongly in favor of their preservation. He hesitated to employ arsenic for the destruction of pulps of deciduous teeth, but had been in the practice of covering the exposed surface with wax, moistened upon one side with creosote, and capping the whole with a metallic filling; several months' experience in such treatment has proven very encouraging.

Dr. Overhalser wished to know whether scattering teeth should be removed before the insertion of an artificial denture.

Dr. Forbes depends upon his own judgment; in cases of chronic periostitis, which he deemed incurable, he would extract, and also where a patient expresses a determination to have a decayed tooth removed and will not listen to reason, he has been prevailed upon to yield.

Dr. Gerry endeavors to save the six-year molars, after exposure of the pulp, but often fails where there exists a strong tendency to local inflammation. He treats periostitis by drilling through the alveolus down upon the apex of the fang, thus drawing blood, and also by depletion of the gums.

Dr. Field extended an invitation for members of the Convention to visit the valuable and interesting museum of Major Cass.

Upon motion, the invitation was accepted, and the Convention resolved to adjourn at 11½ A. M., to reassemble at 3 P. M., in order to afford the members time to avail themselves of this *rare* opportunity for visiting a *rare* collection.

The Chair appointed Dr. Field a committee to wait upon Major Cass, and report the Convention's acceptance of the invitation, and ascertain whether the hour selected would prove convenient.

Dr. Field reported that the hour chosen would prove agreeable.

Upon motion, his report was accepted, and a vote of thanks tendered.

Dr. J. Ward Ellis, in speaking of the instruments employed for extraction, would advocate each one selecting for himself, and using such as he deemed best. He uses cotton, steeped in creosote, for the arrestation of hæmorrhage.

Dr. Whitney uses the perchloride of iron for controlling hæmorrhage.

Dr. Buckingham thought that the remarks of Dr. Ellis in relation to the choice of instruments had covered the ground quite effectually. In extracting, he does not always lance the gum, but makes it a rule to remove a tooth as quickly as possible, and prevent the unnecessary prolongation of a very disagreeable operation. He said that forceps were mostly employed, yet was quite partial to the elevator, and mentioned a dentist of Philadelphia who extracted nine thousand teeth a year, using this instrument exclusively. He had found it more applicable in the lower jaw, particularly for wisdom teeth. Had used it in the upper jaw also, but with less success; it had also proved very serviceable in removing the six front roots. He wants the instrument constructed with an octagonal handle, in order to prevent its turning in the hand upon the application of force.

Dr. Overhalser has used the alveolar cutting forceps with success.

Dr. Allport thought there was a general misapprehension of the force employed in the use of the elevator; many believing that violent pushing or pulling was necessitated. This instrument, however, acts upon the principle of a wedge, a dextrous insinuation of its point between the root and socket accomplishing the removal of the former with the application of but very little force.

Dr. Kulp uses different sizes root forceps, so sharp, that no lancing is requisite.

Dr. Atkinson thought the best instrument, the one that best obeys the will of the operator.

Upon motion, proceeded to the next subject of discussion, "Absorption of the alveolar process—causes and treatment."

Dr. Atkinson said that we must understand general principles in order to treat disease, which is dependent upon one of three causes, viz., psora, psychoses, or syphilis.

Remarked that there were two forms of disease affecting alveolar tissue, viz., mercurial and syphilitic: the former expending its energy upon the periosteum and outer plate at its lower edge, while the latter attacks the cancellous tissue and upper portion of the outer plate, In primary syphilis, the use of creosote, or some application for destroying the action of the virus in its incipiency, is indicated. In the secondary form, constitutional derangements should be first corrected, which he accomplishes by the administration of one grain of hydr. protiodide, and one grain of opii pulv. in pill, until the system is touched with mercury; when the hydriodate of potash is heroically exhibited in a solution formed with iodine. In these cases he remarked that the disease and remedy would counteract each other, the system thus tolerating incredibly enormous doses without the effects of the drug being made manifest. He mentioned a case in which he had gradually increased the dose, from one pill to eight, night and morning, before touching the gums, when he commenced with drachm doses of hydriodate of potash in solution, three times a day. Through this treament the gentleman was enabled to leave at the expiration of six weeks, with the disagreeable odor gone and a good plasm weeping, the appearance of the latter always being an indication for the cessation of treatment. The topical applications which he employs are tinct. iodine, iodine in glycerin, creosote and wine of opium, tannin and glycerin. When the bone is necrossed, he removes the dead portion before the line of demarkation

(*To be continued.*)

